# Abortion reforms in South Africa: An overview of the *Choice on Termination of Pregnancy Act*

**DOI:** 10.4102/safp.v62i1.5240

**Published:** 2020-12-10

**Authors:** Ramprakash Kaswa, Parimalaranie Yogeswaran

**Affiliations:** 1 Department of Family Medicine and Rural Health, Faculty of Health Sciences, Walter Sisulu University, Mthatha, South Africa

**Keywords:** abortion, amendment, counselling, *CTOP Act*, TOP

## Abstract

Access to sexual and reproductive healthcare is a constitutional right and on a broader perspective is part of the universal right to health. The *Choice on Termination of Pregnancy (CTOP) Act* of 1996 was a major step towards commitment to providing comprehensive sexual and reproductive health services in an equitable and rights-based approach. Despite abortion being legally available, unsafe abortion is still an avoidable factor of maternal deaths after more than two decades of abortion law reform in South Africa. The *CTOP Act* 92 of 1996, with its amendments, provides a legislative framework; however, more is needed to reaffirm the sexual and reproductive health freedom.

## Background

South Africa remains committed to providing comprehensive sexual and reproductive health services in an equitable and rights-based approach. Access to sexual and reproductive healthcare is a constitutional right and on a broader perspective as part of the universal right to health. South Africa’s *Choice on Termination of Pregnancy (CTOP) Act* of 1996 was a major step towards achieving sexual and reproductive health freedom.^1,2^ The Act serves as a global role model of reform in the area of abortion laws. South Africa is amongst the top-ranking countries with the most liberal laws on abortions.

The *CTOP Act* of 1996 is manifestly a radical change in the reproductive health sector in South Africa. The *CTOP Act* was passed in November 1996 and first came to effect in February 1997. It replaced the old law of *Abortion and Sterilisation Act* of 1975.^3^ Following this act, abortion-related maternal morbidity and mortality decreased by a dramatic 91% between 1997 and 2002.^4,5^ The 2004 and 2008 amendments reform of the *CTOP Act* of 1996 brought a further reduction in abortion-related maternal morbidity and mortality.^6,7^

Unsafe abortion is a preventable phenomenon and continues to be a major public health problem. Despite abortion being legally available in South Africa after a change in legislation in 1996, barriers to accessing safe abortion services continue to exist.^8^ According to the Saving mothers report, unsafe abortion is still an avoidable factor of maternal deaths after more than two decades of abortion law reform in South Africa.^9^

**FIGURE 1:**
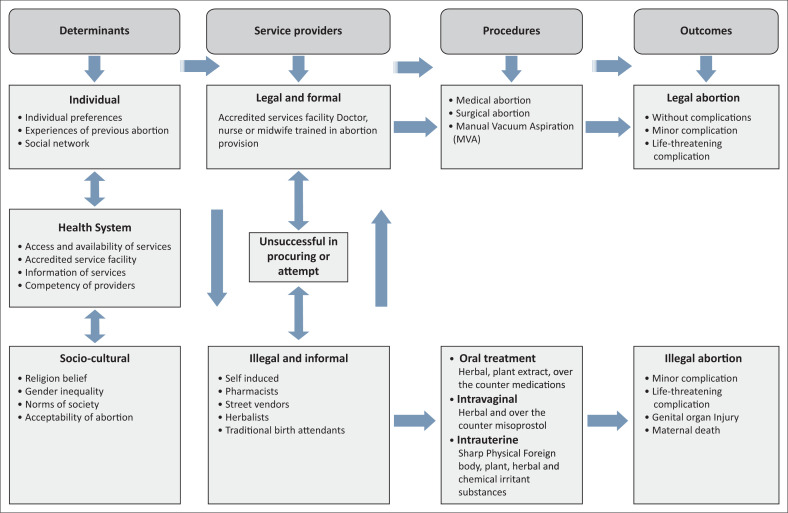
Conceptual framework for women’s abortion-related healthcare needs.

Several studies that have highlighted the barriers to safe abortion in South Africa include provider-related factors such as stigma, provider opposition to abortion, limited knowledge of abortion legislation, unavailability of the services especially in rural areas and lack of technical skill amongst providers because of inadequate training.^10,11^ When the legal abortion services are not assessible or available, women seek help outside the established legal health system, and that brought serious implications on women’s reproductive health and well-being.^12,13^ The conceptual framework for women’s abortion-related healthcare needs is demonstrated in Figure 1. The *CTOP Act* brought a significant improvement in sexual and reproductive health, but barriers to access the safe abortion are still an obstacle to the full benefits of abortion reforms in South Africa.^14,15^

### Circumstances and conditions under which pregnancy may be terminated

The *CTOP Act* enables women of any age to access termination of pregnancy services on request during the first 12 weeks of gestation. Termination of Pregnancy (TOP) is extended up to 20 weeks in certain circumstances where after 20 weeks of gestation under exceptional and life-threatening circumstances with the agreement of at least two medical practitioners.Table 1^3^ summarised the circumstances and timeline for TOP services.

**TABLE 1:** Circumstances and timeline for termination of pregnancy under the *Choice on Termination of Pregnancy Act.*^3^

TOP timeline	Circumstances for TOP	TOP performed by
First 12 weeks and 5 days of gestation	Termination of pregnancy on request	Registered medical practitioner, midwife or nurse (trained for TOP)
13–20 weeks of gestation	Termination of pregnancy available under the following conditions: Rape or incest Danger to a woman’s physical or mental health Foetus not viable Affect woman’s socio-economic status	Registered medical practitioner
Above 20 weeks of gestation	Termination of pregnancy only available under very limited circumstances: Severe threat to the life of woman or foetus Severe foetal congenital problems	Registered medical practitioner

*Source:* Government Gazette. No 17602. The Choice of Termination of Pregnancy, Act 92 of 1996. 1999 [cited 2020 Oct 05];(12). Republic of South Africa. https://www.parliament.gov.za/storage/app/media/ProjectsAndEvents/womens_month_2015/docs/Act92of1996.pdf

TOP, termination of pregnancy.

## Health facility designated for termination of pregnancy

The requirements of the health facility are listed under section 3 of the *CTOP Act* to comply with TOP services. The TOP may take place only at a designated facility and should have access and availability to the following:

- medical and nursing staff
- operating theatre
- surgical equipment
- supplies of intravenous and intramuscular drugs
- emergency resuscitation equipment and an emergency referral centre
- appropriate transport for emergency transfer
- inpatient facilities and equipment for clinical observation
- appropriate infection control measures
- safe waste disposal infrastructure
- communication equipment
- approved by the member of the Executive Council (MEC).

Any health facility with 24-h maternity service and complying with the requirements of section 3 (a) to (j) may be entitled for TOP services up to 12 weeks without having the approval of the MEC. The head of the health facility must notify the relevant MEC that the health facility complies with the requirements of the *CTOP Act*.^3,6^

## Right to information

All women who request TOP services have the right to information concerning the process and procedure of TOP under the *CTOP Act*. The healthcare practitioner with whom the client first requests the TOP service should provide the following information to the client^6^:

- TOP on request during the first 12 weeks and 5 days of the gestation
- TOP from the 13th week and beyond gestation period under certain circumstances
- only her consent is required for the termination of her pregnancy
- non-mandatory and non-directive pre- and post-TOP counselling provision
- options of the TOP procedure (medical vs. surgical)
- alternative options if a woman does not qualify for TOP under *CTOP Act*.

## Counselling

The *CTOP Act* has a provision of non-mandatory and non-directive supportive counselling. Every pregnant woman who is contemplating TOP should be offered pre- and post-counselling from a trained healthcare professional. The counselling should include sufficient information to help women to make their informed choice.^3^

## Pre-termination of pregnancy counselling

Pre-TOP counselling provides the details of the TOP procedure and process. Women have an opportunity to choose the options for TOP available for their gestational age. This also assists women who need emotional support immediately before the procedure. Pre-TOP counselling should include the following^3^:

- the methods of TOP
- pain management options available before, during and after the TOP
- future contraceptive needs and available options
- human immunodeficiency (HIV) counselling and testing services.

## Post-termination of pregnancy counselling

The following information should be provided during post-TOP counseling^3^:

- Oral and written instructions for follow-up care.
- Complications that need medical attention.
- Prescribe healthcare facilities for emergency healthcare needs, if required.
- Offer a contraceptive method and prescription on request.
- When to resume normal activities, including sexual intercourse.

## Consent

**FIGURE 2:**
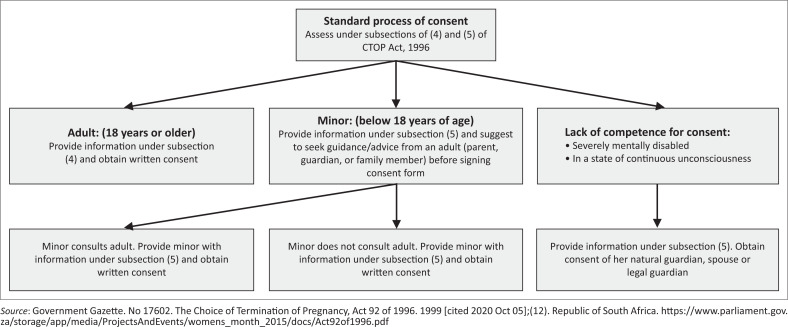
Standard process of consent for termination of pregnancy under the *Choice on Termination of Pregnancy Act 92*, 1996.

The TOP may only take place after the informed consent of the pregnant woman. The *CTOP Act* provides the provision of the consent of pregnant women for TOP regardless of their age.^3^ The *CTOP Act* even allows for minors (< 18 years) to request an abortion without their legal guardian or parents. The healthcare professional is obliged to advise the minor to consult with a parent, guardian or family member; however, the minor may choose or not to do so. Figure 2 summarised the standard process of consent under the CTOP Act.^3^ The constitutional right of a pregnant woman to decide on reproductive health is not age-restricted.^6^

The *CTOP Act* has special provision of the standard procedure with the consent of her natural guardian, spouse or legal guardian or curator under the following circumstances^6^:

- In the case where a woman is severely mentally disabled to such an extent that she is completely incapable of understanding and appreciating the nature or consequences of a termination of her pregnancy.
- In a state of continuous unconsciousness and there is no reasonable prospect that she will regain consciousness in time to request and to consent to the termination of her pregnancy.

## Notification and record keepings

The healthcare facility should maintain health records that are required to complete monthly summary reports containing the number of TOP performed, women’s age group and gestational age. Also, health facilities are obliged to complete an individual notification form for each TOP performed under *CTOP Act*.^6^

## Offenses and penalties

Therefore, in terms of the law, healthcare providers who are not directly involved with the abortion procedure cannot use their beliefs as a reason for not assisting a woman seeking abortion services with information and appropriate referrals. The CTOPA stipulates the following cases as guilty of offense if any person:

- prevents or obstructs access to TOP services terminates a pregnancy by a person who is not a registered medical practitioner or midwife
- allows the TOP at a facility that does not meet the *CTOP Act* regulations.

In terms of the *CTOP Act*, the guilty of an offense is liable on conviction to a fine or imprisonment for a period not exceeding 10 years.^3^

## Third-party certification

The qualification of TOP services is time-restricted under the *CTOP Act* and is particularly challenging because of the health expertise required. The Act leaves considerable scope for interpretation by third-party certification about whether the pregnant woman satisfies the ground for TOP. In the first 20 weeks of pregnancy, there is no third-party certification required. If a woman presented for TOP after the 20th week, then there is a requirement for the medical practitioner to consult with another medical practitioner or midwife about whether the woman meets the ground for abortion.^16^ Furthermore, the *CTOP Act* requires the intervention of two doctors or a doctor and a midwife for them to authorise the TOP if the woman seeking a late-term abortion meets the grounds. This is a form of third-party certification that women need to obtain before accessing the TOP service.^3^

## Conscientious objection

The refusal by healthcare professionals to provide TOP services is often referred to as ‘conscientious objection’, which means ‘to object in principle to a legally required or permitted practice’. The *CTOP Act* does not include any provision explicitly regulating the exercise of conscientious objection. The South African Constitution guarantees the right to freedom of conscience, and implicitly accommodates the right to conscientious objection to TOP in certain circumstances.^16^

This right only applies to the direct provision of services and does not apply to pre- and post-abortion care. Also, the right to conscientious objection would not apply when there is an immediate threat to a woman’s health or her life is at risk.^17^ For example, conscientious objection is always overridden by the healthcare professional’s ethical duty to provide the necessary care during emergencies.

## The choice on termination of pregnancy amendments

The *CTOP Act* of 1996 was amended by Act No. 38 of 2004 and Act No. 1 of 2008. The following changes were amended^6^:

- Empower a MEC to designate facilities that could provide abortion services.
- Exempt a facility providing 24-h maternity services from having to obtain approval for abortion services.
- Provide for the recording of information and the submission of statistics.
- Allow an MEC to make regulations.
- Allow trained registered nurses (not only midwives) to perform first-trimester abortions.

## Discussion

Despite the liberalisation of the abortion law and the relative availability of abortion facilities, access to legal abortion services remains a major challenge for many women in South Africa.^18,19^ As a result of this, there is a high number of illegal abortions performed outside the designated legal facilities. This high number of illegal abortions highlighted that legalisation alone cannot ensure sexual and reproductive health freedom.

Research findings of previous studies in South Africa reported the complex reasons as to why women seek TOPs outside of designated health facilities, which include the lack of knowledge of *CTOP Act*, shortage of public health service providers and denial by healthcare service provider.^10,11^ A lack of knowledge regarding abortion rights under the *CTOP Act* and the perceived poor quality of reproductive health services in the designated facilities are the other important barriers to access the TOP services. The lack of knowledge of the time-limited nature of the TOP services amongst healthcare users and providers forced these disgruntled users to seek help outside the established legal system.^20^ Women seeking TOP services outside the designated facility is associated with higher maternal morbidity and mortality.^8^

Unsafe abortion is a preventable phenomenon and continues to be a threat to sexual and reproductive health in many countries, especially in the developing world.^21^ Despite abortion being legally available in South Africa after a change in legislation in 1996, barriers to accessing safe abortion services continue to exist.^12^ It marks two decades since our landmark legislation was enacted, and women are still demanding the reproductive health rights that the law provides for them. It is a legislative demand that TOP services should be accessible and available to all women in South Africa.

## Conclusion

The CTOP Act 92 of 1996, with its amendments, provides a legislative framework for sexual and reproductive health services, although more work is needed to overcome the barriers to access. In response to mitigate these challenges, there is a need to implement the *CTOP Act* in a standardised and expanded manner, which reaffirmed the sexual and reproductive healthcare as per the constitution.
